# Bowel perforation during enema examination through a colostomy without leakage of contrast agent: A case report

**DOI:** 10.1016/j.ijscr.2020.10.101

**Published:** 2020-10-24

**Authors:** Kaoru Katano, Yuichiro Furutani, Chikashi Hiranuma, Masakazu Hattori, Kenji Doden, Yasuo Hashidume

**Affiliations:** Department of Surgery, Fukui Prefectural Hospital, 2-8-1, Yotsui, Fukui, Fukui, 910-8526, Japan

**Keywords:** UC, ulcerative colitis, CT, computed tomography, Bowel enema, Colostomy, Perforation, Case report

## Abstract

**Introduction:**

Enema examination is considered safe, but in rare cases, complications may result. Here, we report a rare case of iatrogenic bowel perforation during enema examination through a colostomy without leakage of contrast agent.

**Presentation of case:**

A 36-year-old man who had undergone a sigmoid loop colostomy was diagnosed with ulcerative colitis. A bowel enema through a colostomy was performed by nurses and radiological technologists. During the procedure, a balloon catheter was inserted into the proximal lumen of the colostomy, and the balloon was inflated. The patient developed severe abdominal pain a few minutes following withdrawal of the catheter. Computed tomography showed intraperitoneal free air, although contrast agent leakage into the intraperitoneal cavity was not observed. The patient underwent emergency laparotomy. Intraoperatively, there was a 3-cm bowel perforation just inside the colostomy where the inflated balloon was pressing.

**Discussion:**

The perforation site may have been sealed by the inflated balloon during the enema examination. In addition, the patient maintained a supine position during and after the examination. This led to contrast agent accumulating on the dorsal side and not leaking out from the perforation site after the balloon was deflated.

**Conclusion:**

Iatrogenic bowel perforation can occur without leakage of contrast agent during enema examination through a colostomy, and the examination should be performed under the supervision of an attending doctor. In the case of an enema examination through a colostomy, clinicians must be aware of the possibility of bowel perforation even if leakage of contrast agent is not observed.

## Highlights

• Enema examination is considered safe, but in rare cases, complications may result.
• Iatrogenic bowel perforation can occur without leakage of contrast agent during enema examination through a colostomy.
• In the case of an enema examination through a colostomy, clinicians must be aware of the possibility of bowel perforation even if leakage of contrast agent is not observed.

## 1 Introduction

Generally, bowel enema is considered safe, but in rare cases, complications may result [1]. Bowel perforation is a life-threatening complication of this examination, usually diagnosed by the leakage of contrast agent. Here, we report a rare case of iatrogenic bowel perforation during enema examination through a colostomy without leakage of contrast agent.

This manuscript has been reported in line with the SCARE guidelines [2].

## 2 Presentation of case

**Fig. 1:**
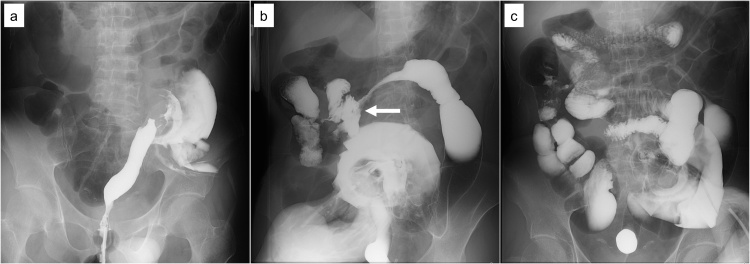
Enema examination findings. **a:** Enema examination through the anus revealed no abnormal findings. **b:** Enema examination through the colostomy revealed a fistula between the transverse colon and small intestine (white arrow). **c:** Leakage of contrast agent was not observed.

**Fig. 2:**
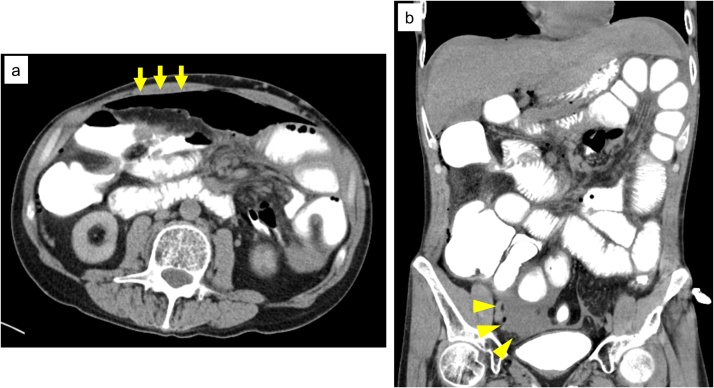
CT findings. CT showed intraperitoneal free air (**a:** yellow arrows) and a small amount of ascites (**b:** yellow arrowheads). Leakage of contrast agent into the intraperitoneal cavity was not observed.

**Fig. 3:**
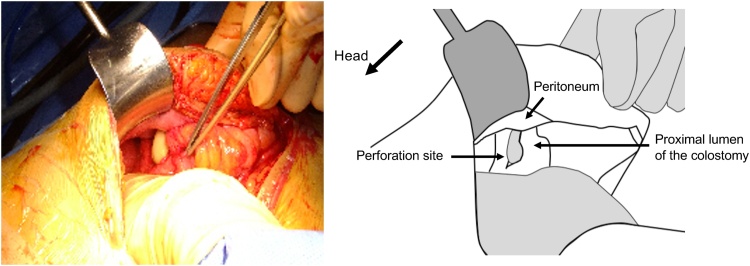
Intraoperative findings. The index finger of the right hand was inserted into the proximal lumen of the colostomy. There was a 3-cm bowel perforation just inside the colostomy.

A 36-year-old man was diagnosed with pancolitis-type ulcerative colitis (UC) and had been treated with mesalazine and steroids for approximately three years. He was scheduled for proctocolectomy because endoscopic examination with biopsies revealed high-grade dysplasia. He had undergone a sigmoid loop colostomy for management of a perianal abscess three and a half years ago. To assess the intestinal fistula, a bowel enema was performed by nurses and radiological technologists. First, Gastrografin was introduced through the anus, which revealed no abnormal findings. Then, enema examination through a colostomy was performed. During the procedure, a balloon catheter was inserted into the proximal lumen of the colostomy, and the balloon was inflated with 20 mL of air. The enema examination revealed a fistula between the transverse colon and small intestine, while leakage of contrast agent was not observed (Fig. 1). The patient developed severe abdominal pain a few minutes following withdrawal of the catheter, and abdominal computed tomography (CT) was performed. CT showed intraperitoneal free air and a small amount of ascites, although contrast agent leakage into the intraperitoneal cavity was not observed (Fig. 2). The patient underwent emergency laparotomy the same day. Intraoperatively, there was a 3-cm bowel perforation just inside the colostomy where the inflated balloon was pressing (Fig. 3), and other perforation sites were not observed. The stoma was pulled out, resulting in a perforation site outside the abdominal wall. He did not have any complications and was discharged from our hospital 14 days after the surgery.

## 3 Discussion

This case revealed two important clinical suggestions. First, iatrogenic bowel perforation during enema examination through a colostomy can occur without leakage of contrast agent. Bowel perforation is the most frequent serious complication of this examination, occurring in approximately 0.02% to 0.04% of patients [1]. Injury to the intestinal mucosa due to the enema tip or retention balloon is probably the most common traumatic cause of this complication [1]. In particular, the risk of bowel perforation is high in the case of an enema examination through a colostomy [3]. In the present case, the inflated balloon was most likely the cause of the perforation, as the perforation site was located just inside the colostomy where the inflated balloon was pressing. Impairment of the tensile strength of the bowel wall due to UC and long-term steroid therapy was thought to have contributed to the perforation. However, the enema examination did not reveal leakage of contrast agent, and thus, we did not initially suspect bowel perforation. Our patient developed severe abdominal pain a few minutes following withdrawal of the catheter, while CT scans revealed intraperitoneal free air. We can assume the following two reasons why our patient did not show contrast agent leakage. First, the perforation was caused by the inflated balloon. The perforation site may have been sealed by the inflated balloon during the enema examination. Second, the patient maintained a supine position during and after the examination. This led to contrast agent accumulating on the dorsal side and not leaking out from the perforation site after the balloon was deflated. There are a few reports of bowel perforation during enema examination through colostomy, as the incidence is so low [4]. To the best of our knowledge, similar cases have not been reported in the literature.

The second clinical suggestion is that bowel enema through a colostomy should be performed under the supervision of an attending doctor. In the case of enema examination through a colostomy, the anatomy of the stoma and weakness of the intestinal tract wall near the stoma differ according to the patient. Advice from the primary surgical team should be sought to avoid complications. The literature suggests that if there is doubt about disease at the stoma, balloons should not be inflated inside the stoma to avoid injury to the intestinal mucosa [5]. If inflation of the balloon is contraindicated, inspectors should inflate the balloon outside the stoma and push the inflated balloon against the outside of the stoma to seal the stoma opening [5]. In the present case, we must admit that we should not have inflated the balloon because impairment of the bowel wall near the colostomy was suggested. To reduce this complication, clinicians must be aware of the hazard of inflating balloons inside the stoma.

## 4 Conclusion

Iatrogenic bowel perforation during enema examination through a colostomy can occur without leakage of contrast agent and bowel enema through a colostomy should be performed under the supervision of an attending doctor. Although bowel perforation during enema examination is rare, delayed diagnosis may lead to poor outcomes. In the case of an enema examination through a colostomy, clinicians must be aware of the possibility of bowel perforation even if leakage of contrast agent is not observed.

## Declaration of Competing Interest

The authors declare that they have no competing interests.

## Funding

This study was not funded.

## Ethical approval

Ethical approval for this report has been exempted by our institution.

## Consent

Written informed consent was obtained from the patient for publication of this case report and accompanying images. A copy of the written consent is available for review by the Editor-in-Chief of this journal on request.

## Author contribution

KK wrote the manuscript and prepared the manuscript under the supervision of YF. KK, YF and KD performed the surgery. Other co-authors discussed the content of the manuscript. The authors read and approved the final manuscript.

## Registration of research studies

1. Name of the registry: Research Registry
2. Unique identifying number or registration ID: researchregistry6088
3. Hyperlink to your specific registration (must be publicly accessible and will be checked): https://www.researchregistry.com/browse-the-registry#home/registrationdetails/5f7d8d67c3666000150d74fa/

## Guarantor

Kaoru Katano, corresponding author of this article.

## Provenance and peer review

Not commissioned, externally peer-reviewed.
